# Substrate mediated interaction between pairs of keratocytes: Multipole traction force models describe their migratory behavior

**DOI:** 10.1371/journal.pone.0212162

**Published:** 2019-03-01

**Authors:** Benoit Palmieri, Christine Scanlon, Daniel Worroll, Martin Grant, Juliet Lee

**Affiliations:** 1 Department of Physics, McGill University, Montréal, Québec, Canada; 2 Department of Molecular & Cell Biology, Storrs, CT, United States of America; The University of Akron, UNITED STATES

## Abstract

A series of traction force microscopy experiments involving pairs of keratocytes migrating on compliant substrates were analyzed. We observed several instances where keratocytes that are about to collide turn before they touch. We term this phenomenon *collision avoidance behavior* and we propose that the turning is caused by the substrate mediated elastic interactions between the cells. A multipole analysis of the cell traction reveals that the left-right symmetry of the keratocyte traction pattern is broken during collision avoidance events. The analysis further shows that the cell migration direction reorients *before* the principal traction dipoles as the cells turn. Linear elasticity theory is used to derive the cell-cell interaction energy between pairs of keratocytes. The traction force applied by each cell is modeled as a two points (dipole) or three points (tripod) force model. We show that both models predict that cells that are about to collide in a head-on manner will turn before touching. The tripod model is further able to account for the quadrupole components of the traction force profile that we observed experimentally. Also, the tripod model proposes a mechanism that may explain why cells tend to scatter with a finite angle after a collision avoidance event. A relationship between the scattering angle and the traction force quadrupole moment is also established. Dynamical simulations of migrating model cells are further used to explain the emergence of other cell pair trajectories that we observed experimentally.

## Introduction

The ability of cells to reorient in response to changes in the physical properties of their environment is well known [1, 2]. Capillary endothelial cells will reorient perpendicular to applied strain [3], and cells attached to flexible surfaces exhibit durotaxis [4], in which they move towards regions of increased rigidity. Cancer metastasis is also promoted by the tendency of abnormal cells to migrate towards stiffer regions of the extracellular matrix (ECM) at the edge of tumors [5]. Most of the recent research emphasis has been on the reorientation of cells in sheets to external stresses [6] or the guidance cues provided by substrate stiffness [4, 5]. However, there is evidence that cells can respond to the mechanical signals transmitted via the substrate by their neighbors without direct contact. For example, recent studies have shown that bovine aortic endothelial cells extend a pseudopod toward a neighboring cell, when attached to a surface of intermediate stiffness [7]. Therefore, it is possible that the direction of cell movement is influenced by the forces that a neighboring cell transmits through the substrate. The goal of the following study is to investigate this possibility by performing traction force microscopy (TFM) with pairs of fish epithelial cells (keratocytes) as they approach in close proximity to each other and to explain the observed behavior with a simple theoretical model.

Keratocytes are uniquely suited for this study. Firstly, they exhibit a rapid “gliding” mode of movement, while maintaining their shape, speed and direction for many minutes at a time [8]. Secondly, the traction force pattern has been characterized in which the highest forces are localized at the lateral rear edges, and low tractions are found at the front [9]. Finally, keratocytes are mechanosensitive, and respond both to forces generated intracellularly and to externally applied stresses such as local substrate indentation using a microneedle [10].

To determine whether keratocyte movement is influenced by the traction stresses generated by a neighboring cell, we observed the motile behavior of approaching pairs of keratocytes attached to two substrates of different stiffness. The two substrates were 3.5% and 10% gelatin gels, with corresponding Young’s moduli of ≈1–2 kPa and ≈7 kPa, respectively. We found that approaching pairs of cells would often begin to turn away from each other without touching, in what we term *collision avoidance* behavior. This phenomenon is more easily observed on the softer substrate. On the stiffer one, cells that are about to collide usually do.

We rationalize the emergence of collision avoidance behavior by constructing a minimum energy model that treats cells as self-propelled multipoles. In our model, each keratocyte is represented by a two or three point force distribution model (refered to as the dipole or tripod models) where the distance between each point is of the order of the cell size and accounts for most collision avoidance occurrences. Force dipole models for cells have first been proposed in Ref. [11]. Both models reproduce the traction dipole moments that we measured experimentally and the tripod model can further reproduce the quadrupole moments. For two approaching cells to scatter (without touching) with a finite angle between them, the theory shows that they must have a non-zero traction force quadrupole moment. The dipole force model encourages the cells to synchronize their motion and migrate together in a side-by-side fashion. We have not observed such a behavior in any of our experiments.

Dynamical simulations of the model were also performed. These are characterized by the introduction of two distinct timescales: 1) the first arises from cell velocity and 2) the other arises from cell reorientation in response to the substrate mediated elastic interactions. Simulation of cell pair trajectories where these timescales and the initial conditions (cell position and orientation) are varied shows how numerous types of migratory behavior can emerge. Some of these simulated behaviors have been observed in our experiments. In short, combining our experiments with a simple theoretical model allows us to match the parameters of the model with the observed collision avoidance behavior. Different migratory behaviors are predicted to emerge for varying parameters such as the cell velocities, their tendency to reorient, the relative strength of the dipole/quadrupole moments and the two time scales described above.

The paper is divided as follows. The experimental procedure and the theoretical model are detailed in the Materials and methods section. The first part of the Results section summarizes the experimental observations of *collision avoidance* behavior and shows how various force multipoles can be extracted from traction patterns obtained from the displacements of beads embedded in the substrate. The second part introduces the dipole and tripod cell models which are compared with the most significant force multipoles observed in the experiments. Further, the substrate-mediated interaction energy landscape for two cells modeled as tripods and dipoles is reported and used to explain how collision avoidance arises. The results of this subsection are obtained in the limit where the timescale for reorientation is infinitely small compared with the one that characterizes forward cell motion. Finally, in the third part of the Results section, we move away from this regime and use a simple dynamical model to simulate the migratory behavior of cell pairs that interact through the substrate. We report a number of cell pair trajectories that emerge when parameters such as cell velocity and resistance to reorientation are varied. The theoretical results obtained are tied together with the experimental results at the end of the Results section and in the Conclusion.

## Materials and methods

### Experimental details

#### Cell culture

Fish epithelial keratocytes were cultured from Molly fish Poecillia sphenops scales, as described previously [10]. Scales were obtained from live Molly fish by gently removing about 10 scales per flank with a pair of fine forceps. After 2 − 3 days in culture cells were dissociated and re-plated onto a gelatin gel contained within a glass chamber containing 400 *μl* RPMI medium supplemented with (2%) serum.

#### Preparation of gelatin substrata

Gelatin gels were prepared as described previously [12]. Briefly, stock gels of 3.5% or 10% unflavored Knox gelatin (Nabisco, Parsippany, NJ) dissolved in Ca^2+^ and Mg^2+^ free PBS were prepared and stored as 5 *ml* aliquots at 4°C until required. Before use, the gel was liquefied and a 400 *μl* aliquot was transferred into a glass Rappaport chamber then allowed to solidify at 4°C. A solution of fluorescent microspheres at a concentration of 1:100 (0.5 *μm*, Invitrogen) in distilled water was used to coat the surface of the gel for ≈ 30 seconds, before being aspirated off and dried for about 1 hour, at 4°C. Prior to cell attachment, gels were placed briefly on a hot plate to liquefy the lower layer of gelatin, so that 330 *μl* of it could be carefully aspirated off. This resulted in a ≈ 40 *μm* thick layer of gelatin whose top surface was embedded with a monolayer of fluorescent beads. Gels were calibrated using the microsphere indentation method, as described previously [13]. The Young’s moduli of 3.5% and 10% gelatin gels were ≈ 1–2 kPa and ≈ 7 kPa, respectively.

#### Imaging

To monitor collision frequency between cell pairs moving on soft or stiff surfaces, phase contrast microscopy was performed on a Nikon Eclipse TE300 microscope using an ×20 objective. Images were acquired every 10 seconds for 30 minutes, with a Quantix 57 CCD camera controlled by ISee Imaging software. For traction force microscopy, paired Leica modulated contrast (LMC) images of cells and fluorescence imaging of marker beads in the substratum was performed on an inverted Leica DMIRB microscope, using an ×40 LMC objective. Images were acquired every 10 seconds for 30 minutes with a Photometrics Coolsnap (Roper Scientific) HQ camera controlled by Metamorph for Olympus software.

#### Cell morphometry

Cell outlines were obtained from LMC images by tracing along the cell edge using the “polygon” tool in ImageJ, followed by the “fit spline” function. Cell outlines were used to generate binary images (or masks) of the cell area, from which all other morphometric data were obtained. Cell turns were identified from plots of the cell centroid as a change in angular motion that was at least 15 degrees in magnitude. Difference images were generated by subtracting the current image from the next, for a complete sequence of images. These were used to aid detection of the cell edge, and for determining when the substratum deformation fields of two approaching cells begin to overlap. The deformation field is defined here as the region that encompasses the smallest observable bead displacements (≈0.5 *μm*) associated with a single cell. These appear as black and white stipples in the difference image and can extend up to ≈7 *μm* from the cell margin. Deformation fields are considered to be overlapping when the bead displacement field from one cell cannot be distinguished from that of its neighbor.

#### Traction force microscopy

Calculation of the traction stresses generated by moving keratocytes, and plots of traction vector maps were made using the LIBTRC traction analysis software written and developed by Micah Dembo [14, 15]. Other types of approaches could have been used such as the one proposed in Ref. [16].

### Theoretical details

#### Elastic interaction between two tripods

The substrate mediated interaction energy between two keratocytes can be obtained by describing the substrate as an elastic half-space. This uses the approximation that the substrate is thick enough so that the boundary conditions at the gel/glass interface can be neglected [17]. Given that two cells apply traction **t**_1_(**x**) and **t**_2_(**x**) on the substrate, their interaction energy can be written as,

$$\begin{array}{c} F_{1,2}=\int dx\int dx^{\prime }\;t_{1}\left(x\right)G\left(x-x^{\prime }\right)t_{2}\left(x^{\prime }\right), \end{array}$$ (1)

where G(**x** − **x**′) is the Green’s function for the elastic half-space given by [18],

$$\begin{array}{c} G\left(x\right)=\frac{1+\sigma }{\pi E}(\begin{array}{cc} \frac{1-\sigma }{r}+\sigma \frac{x^{2}}{r^{3}} & \sigma \frac{xy}{r^{3}}\\ \sigma \frac{xy}{r^{3}} & \frac{1-\sigma }{r}+\sigma \frac{y^{2}}{r^{3}} \end{array}), \end{array}$$ (2)

where $r=\sqrt{x^{2}+y^{2}}$ and where *E* and *σ* are, respectively, the Young’s modulus and Poisson ratio of the elastic substrate. Note that here, for simplicity and to keep the final expression tractable numerically, it is further assumed that the traction has no component normal to the substrate plane. This level of approximation was used in many recent studies [19]. Note that, throughout the text, a bold face variable denotes a vector in the 2D plane. For example, $t=t_{x}\hat{x}+t_{y}\hat{y}$ where *t*_*x*(*y*)_ are the components along the directions of the unit vectors $\hat{x}\left(\hat{y}\right)$.

Closed form expression for the interaction energy between cells can therefore be obtained from Eq (1) by assuming simple models for the distribution of traction applied by each cell. For example, treating cells as force dipoles allows one to predict the most favorable orientation between a pair [19] or a group of cells [20, 21].

From now on, we will set the Poisson ratio of the substrate to 1/2, unless specified otherwise, which means that we treat the substrate as an incompressible medium. This is a good approximation for the gels used in the experiments. Examples of the effects the Poisson ratio can have on cell-cell interactions where the cells are modeled as point force dipoles are given in Ref. [20]. Further, we assumed that the deformation of the substrate is small enough so that the linear elasticity theory can be used. For an example of non-linear elasticity effects of the substrate in a single cell system, see Ref. [22]. These considerations go beyond the scope of the present study.

Under this condition, S1 Appendix gives the complete interaction energy between a pair of cells modeled as tripods; the energy is obtained by inserting an equation of the form of Eq (6) for **t**_1_ and **t**_2_ in Eq (1). The results reduce to the dipole interaction energy in the limit *β* → 0.

## Results

### Experimental results

**Fig 1:**
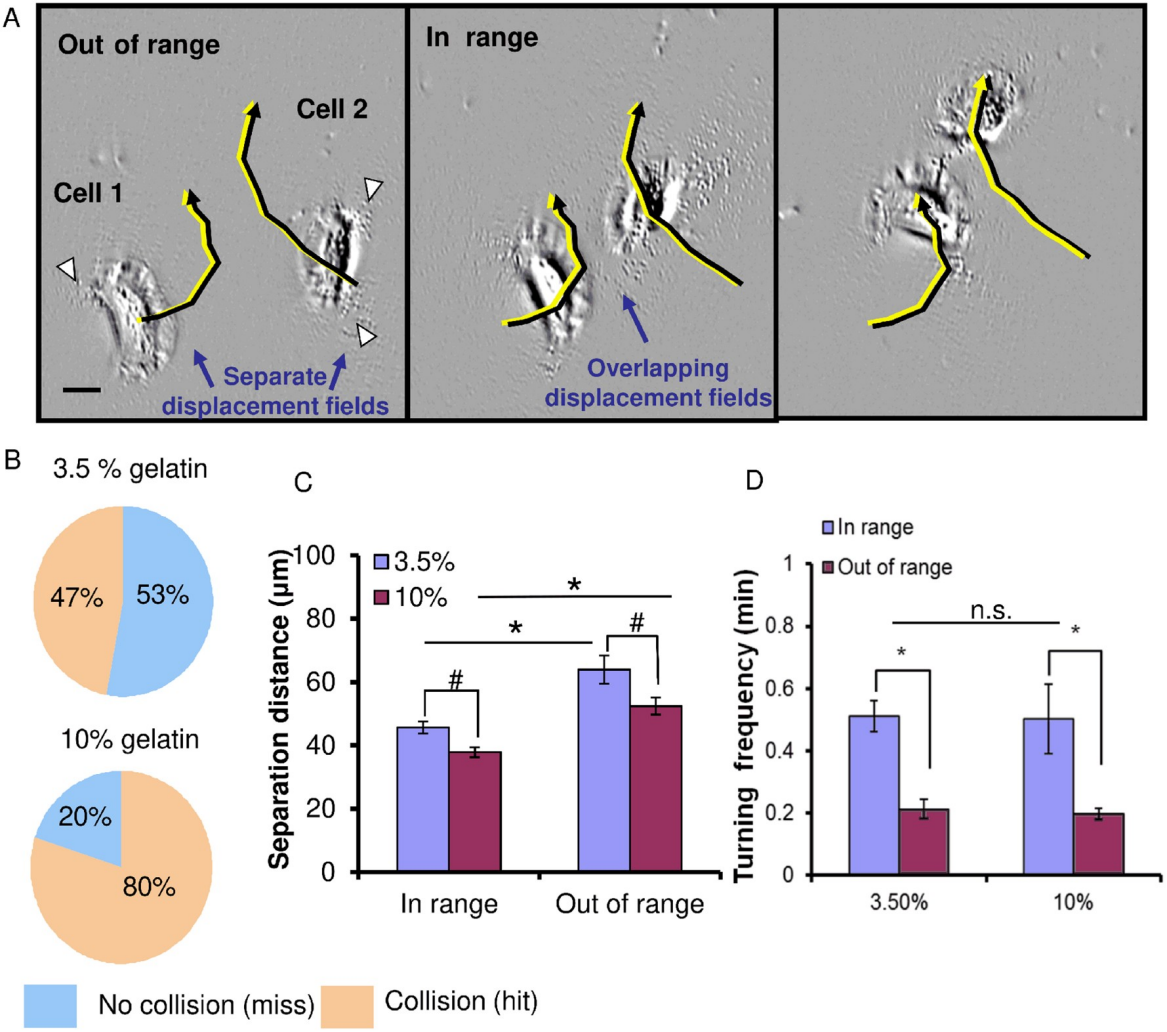
Part A. A pair of approaching cells displaying collision avoidance on a soft substrate. Time series of difference images of two cells is shown at 0, 3.3 and 6.7 minutes, respectively, as they move along the paths indicated (black and yellow lines). Initially, cell 1 (left) and cell 2 (right) are approaching each other but their bead displacement fields do not yet overlap. Bead displacements are proportional to substrate deformation and appear as black and white stippled areas at the rear, left and right cell edges (white arrow heads). At 3.3 minutes cell deformation fields start to overlap as cells are in range, and begin to turn away from each other. At 6.7 minutes cells are still in range but they are moving apart. Scale bar is 3.3*μm*. Part B. The effect of surface stiffness on collision avoidance. Pie charts show the percentage of cells that collide (hit) or undergo collision avoidance (miss) on softer, 3.5% and stiffer, 10% gelatin gels. Part C. Histogram showing the average cell-cell separation distance at which the displacement fields of each cell first overlaps (in range) and separate (out of range) on softer (3.5% gelatin) and stiffer (10% gelatin) surfaces. For cell pairs in range, *n* = 60 (softer) and *n* = 23 (stiffer). For cell pairs out of range, *n* = 28 (softer) and *n* = 67 (stiffer). Part D. Histogram showing the frequency of turns when cells are in range or out of range, on softer (*n* = 33, in range; *n* = 16, out of range) and stiffer surfaces (*n* = 17, in range; *n* = 16, out of range). Data in B-D were obtained from 8 independent trials. * Denotes significance using a Wilcoxon signed rank test, where *p* ≤ 0.001. # Denotes significance using a Mann-Whitney U test, where *p* ≤ 0.01. Error bars represent S.E.M.

The emergence of collision avoidance is shown in Fig 1 where two moving cells approach each other, initially in a “head-on” configuration. Later, we observe that the substrate displacement fields produced by each cell start to overlap. At that point, each cell turns, or scatters away from the other, without touching. This behavior appears to be influenced by substrate stiffness (Fig 1B) since collision avoidance was more frequent on the softer gels (≈ 1 − 2 kPa) with 53% of approaching cell pairs avoiding collision, compared with only 20% on the stiffer gels (≈7 kPa). This suggests that neighboring cells “sense” the proximity of each other sooner on softer gels. Consistent with this, we find that the distance at which the bead displacement field of each approaching cell begins to overlap (in range) is greater on softer gels compared with stiffer ones, ≈45 *μm* and 37 *μm*, respectively. Similarly, the distance at which this combined bead displacement field separates into two (out of range) is greater (≈65*μm* compared with 50*μm*) on the softer versus the stiffer gels, respectively (Fig 1C). To determine whether cell proximity affects cell turning behavior, we measured the turning frequency of cells when in or out of range. We found that the turning frequency was significantly increased when cells were in range on both the softer and stiffer gels (Fig 1D). It is noteworthy that the in range and out of range values for cell turning frequency was the same for cells on the two types of gels, indicating that the mechanism of cell turning is unaffected by substrate stiffness. The upcoming analysis proposes that collision avoidance emerges due to substrate mediated cell-cell interaction.

**Fig 2:**
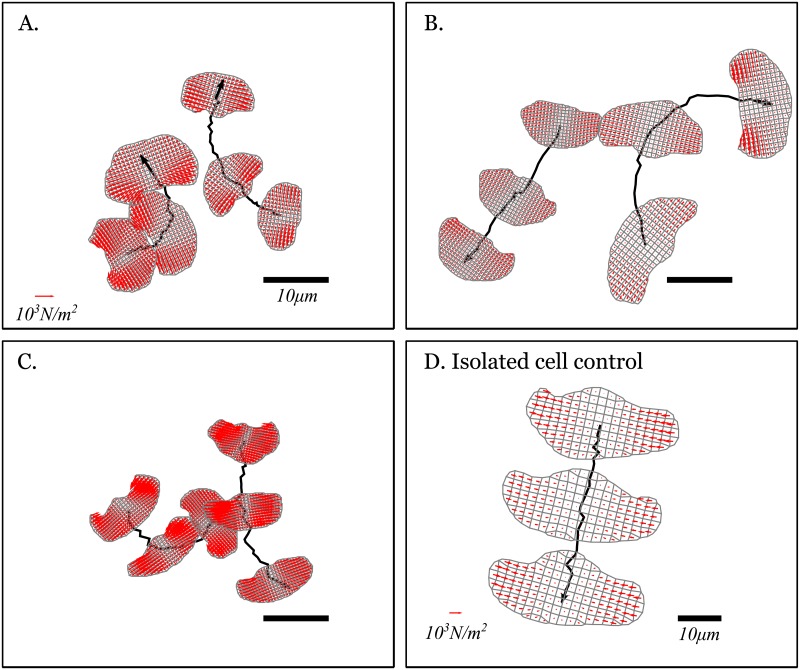
Trajectories of migrating keratocytes with their underlying traction map. Parts A-C: Cell turning induced by substrate mediated cell-cell interactions. Part D: An isolated cell moving from the top to the bottom of the observation region. In all panels, the cell contours and the traction force that both cells apply on the substrate (Red arrows) are shown. Within the same panel, the arrow length is indicative of the local traction force magnitude. The traction force was calculated from the analysis of substrate embedded bead displacements and linear elasticity theory [15, 18]. The traction is constrained to vanish outside the area covered by the cells and is calculated using an irregular mesh (shown in Gray). Note that the scale differs from Part D compared to the other panels, which are on the same scale. A red arrow in the bottom left of panels A and D indicate a traction equal to 10^3^ *N*/*m*^2^ and a black bar in the bottom right indicates a length equal to 10 *μm*. The final position of the cells is overlayed with an arrowhead and the total duration of the trajectories are 500, 250, 310 and 310 seconds in parts A, B, C and D, respectively. The traction maps for cell pairs undergoing collision avoidance represent 3 of 6 cells. The isolated cell is one of 3 whose tractions were mapped. Accompanying movies for the four panels are given in S1, S2, S3 and S4 Videos.

We now focus on three individual cell pair trajectories where the migration path appears to be influenced by substrate mediated interactions. Fig 2 shows these cases along with a typical isolated migrating cell control. The traction forces that each cell exerts on the substrate are calculated at each time frame and are shown as red arrows in Fig 2. The first three panels show pairs of keratocytes that approach one another and where their substrate mediated interactions induces turning for one or both cells, thereby preventing them to touch. The first case shown in panel A is the clearest example of collision avoidance behavior (also shown in Fig 1A without showing the traction force vectors). Both cells move toward one another at about the same speed in a nearly head-on fashion and they both turn and scatter away from one another also without touching. In the second case (panel B), only one of the two cells turn and in the third case (panel C), one of the two cells turns behind the other, faster moving, cell. Panel D shows an isolated keratocyte moving in a linear fashion. Isolated keratocytes can undergo turning behavior, but these are usually separated by long and straight trajectory segments such as the one shown. Accompanying movies for the four panels of Fig 2 are given in S1, S2, S3 and S4 Videos.

We first analyze in more detail the clearest case of collision avoidance behavior shown in Fig 2A. This is done by computing various traction force integrals from the experimental data. The first one gives the magnitude of the traction force:

$$\begin{array}{c} T_{n}=\int_{A_{n}}dx\;\left|t_{n}\left(x\right)\right|, \end{array}$$ (3)

where the integral over **x** is performed over the area under cell *n*, *A*_*n*_. Also, **t**_*n*_(**x**) is the traction (force per area) exerted by cell *n* at point **x**. Note that the traction force calculation was performed with the constraint that the total force applied by a cell on a substrate [i.e., the monopole component, $\int_{A_{n}}dx\;t_{n}\left(x\right)$] vanishes. In principle, the cell needs to apply a net force, or non-zero monopole, to move against the environment viscous forces. However, this force is very small compared to the higher multipole components. Hence, the distribution of forces is characterized by higher force moments. Further, it was assumed that the distribution of forces is constrained to lie within the area covered by the cell (the Cell Morphology subsection explains how the cell contour was determined).

The dipole is the next non-vanishing traction force moment integral,

$$\begin{array}{c} D_{n,ij}=\int_{A_{n}}dx\;x_{i}t_{n,j}\left(x\right), \end{array}$$ (4)

where *i* and *j* can be either of the 2D spatial components (*x* and *y*). The next non-vanishing traction force integral moment is the quadrupole,

$$\begin{array}{c} Q_{n,ijk}=\int_{A_{n}}dx\;x_{i}x_{j}t_{n,k}\left(x\right). \end{array}$$ (5)

The quadrupole moment is less commonly employed to characterize cell traction. However, a recent study on migrating *Dictyostelium Discoideum* showed that it is strongly correlated to the cell direction of motion [23]. We will show that the quadrupole moment plays an important but different role in our study as well. The dipole force integral is a 2×2 matrix. Its eigenvalues and eigenvectors respectively represent the two principal dipole moments and their orientations. The quadrupole force integral has three indices. In what follows, we will report the various components of *Q* in the basis where *D* is diagonal. More precisely, the subscript of *Q* will be designated by *a* and *b* which are, respectively, the axis of the large (*λ*_*a*_) and small (*λ*_*b*_) principal dipoles moments.

**Fig 3:**
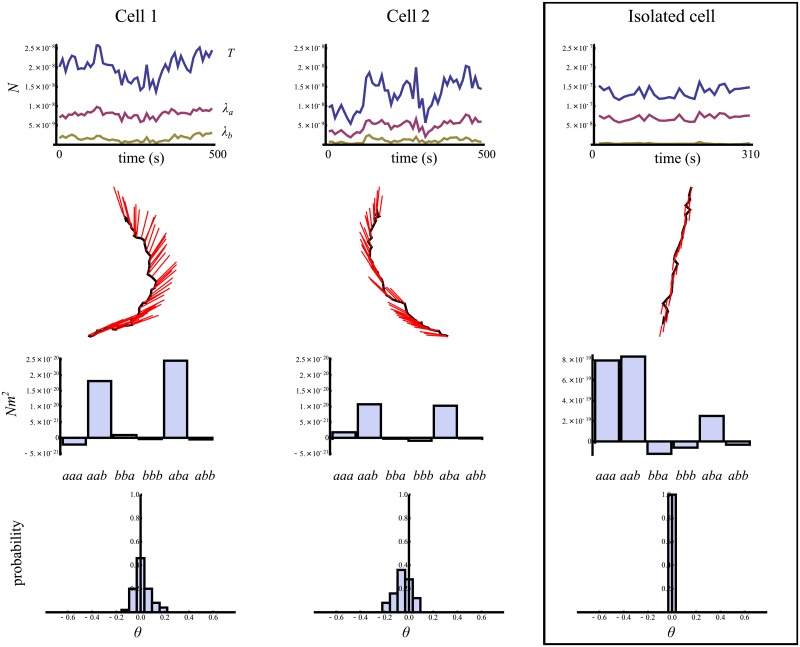
Traction force analysis of the two interacting cells shown in Fig 2A (left 2 columns) and the isolated cell shown in Fig 2D (right column). The top panels give the amplitude of the traction magnitude, in blue (*T*), and the two principal force dipole moments (divided by the square root of the mean cell area), in magenta (*λ*_*a*_) and yellow (*λ*_*b*_), as a function of time. The second row of panels shows the small dipole orientation (red lines) at fixed time intervals along the cell trajectories (black arrow). The third row shows the observed distribution of the angle between the small dipole and the short cell shape eigenvector. The last row gives the strength (average over the cell trajectory) of the 6 independent quadrupole moments, as explained in the main text and in Eq (5). A sketch of a simple model for the distribution of traction forces within the cell that accounts for the most important quadrupole moment, *aab*, is shown in the left panel of Fig 4. As explained in the text and in S1 Appendix, the other large quadrupole components are trivial corrections to a perfect dipole. In the figure, *N* and *m* are the SI units of force and length.

Fig 3 summarizes the time dependence and time average quantities of the cells trajectories shown in Fig 2A and 2D. The top row shows the cell traction and the large and small principal dipole moment of all cells (colliding pair of cells and isolated control) as a function of time. This illustrates the fact that for keratocytes, one of the two dipoles is much larger than the other. Note the different scale used for the pair of cells versus the isolated one. In fact, the latter larger cell applies much larger traction on the substrate. The panels in the second row shows an overlay of the *smallest* dipole component vector (red lines) with the cell trajectory. As expected for keratocytes [9, 24], the small dipole is parallel to the cell trajectory for the isolated control. However, this alignment is absent for the interacting cell pair. In this case, the left and middle panels show that the cell migratory direction responds more rapidly to the presence of the other cell. In other words, the collision avoidance is characterized by a change in migratory direction *followed by* a reorientation of traction force applied by the cell.

The third row shows histograms of the angle between the long cell axis and the orientation of the largest principal dipole. The long cell axis is obtained by an eigenanalysis of Eq (4) with the traction force vector (**t**) replaced by the position vector inside the cell (**x**). Unsurprisingly, the largest force dipole is aligned with the cell’s long-axis for the isolated control. In the other two cases, the angle observed between the two vectors significantly deviates from zero. Further, the two distributions have longer tails in the positive (negative) angles direction for the left (right) cell. This results in a net (and opposite in sign) non-zero mean angle between the cell shape axes and the traction force axes. This is one way to characterize the momentary loss of traction force left-right symmetry when the cells are in close proximity. The observation that the average angle has opposite signs for the left and right cells is simply due to the fact that the two cells turn in opposite directions.

The last panel of Fig 3 shows the average magnitude of all Quadrupole components. There are six independent components. The figure clearly shows that, in all cases, three of these components dominate the others. These are *Q*_*aaa*_, *Q*_*aab*_ and *Q*_*aba*_. S1 Appendix shows that *Q*_*aaa*_ and *Q*_*aba*_ are trivial corrections to a perfect dipole; they do not break the symmetry of the force dipole. On the other hand, *Q*_*aab*_ has a more profound meaning. In the following section, we show how this quadrupole moment plays an important role in the collision avoidance behavior. The traction force analysis of the other cell pair trajectories shown in Fig 2(B) and 2(C) are given in S1 Appendix.

### Dipole and tripod force models: Static considerations

**Fig 4:**
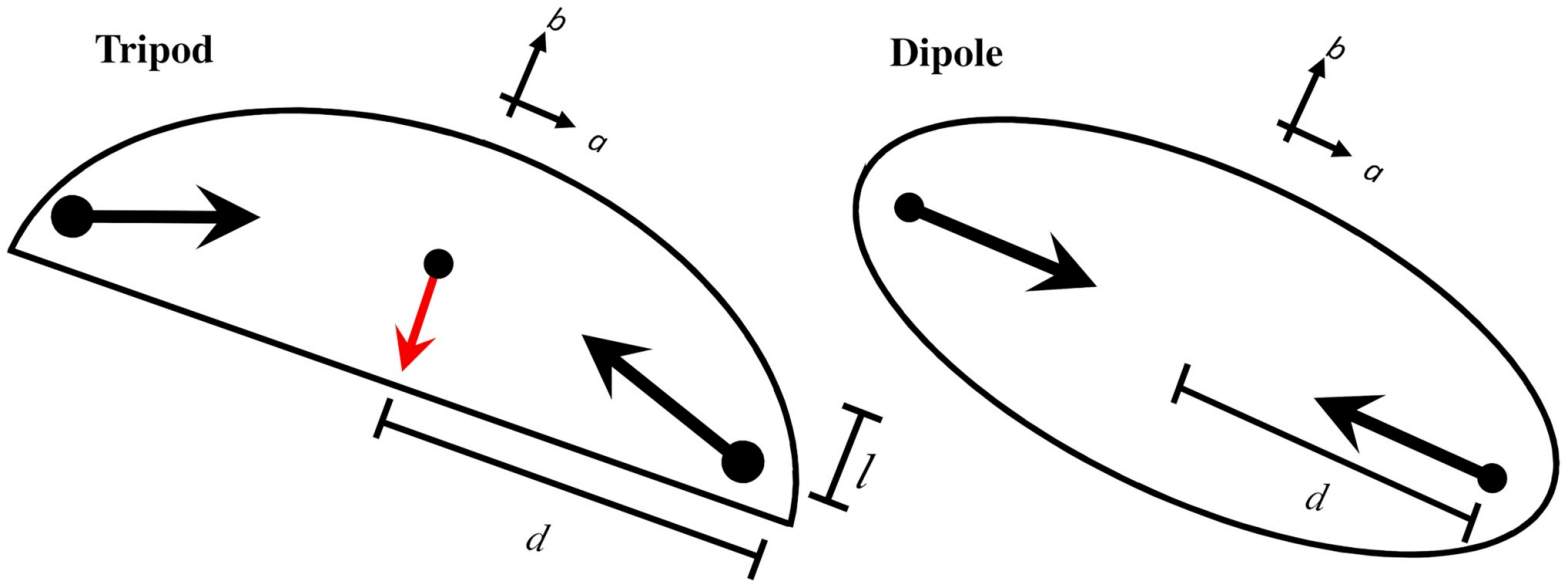
Sketch of the tripod force model and the dipole force model. In both cases, large opposing tractions are oriented along *a* which leads to a dominant traction dipole moment in that direction. In the tripod model, smaller tractions are oriented along *b*. In both cases, the net traction integrated over the cell area vanishes. The arrangement of the tractions for the tripod model generate a net quadrupole moment, *Q*_*aab*_, whereas all quadrupole moments vanish for the dipole model. The strength and relative orientation between the 2 or 3 point traction vectors are exaggerated, for clarity. Distances mentioned in the text, *d* and *l*, are shown in the figure. The cell contour is strictly illustrative.

We now propose simple models for the traction force distribution inside each cell that accounts for the most important traction force moments mentioned above. The left/right side of Fig 4 shows the three points(tripod)/two points (dipole) force distribution models we propose:

$$\begin{array}{ccc} t=\\ & & \hat{a}(\alpha \delta \left(x+\hat{a}d\right)-\alpha \delta \left(x-\hat{a}d\right))\\ & & +\hat{b}(\frac{\beta }{2}\delta \left(x+\hat{a}d\right)+\frac{\beta }{2}\delta \left(x-\hat{a}d\right)-\beta \delta \left(x-\hat{b}l\right)) \end{array}$$ (6)

where the orientation of the large (small) dipole component is parallel to the unit vector $\hat{a}$ ($\hat{b}$). 2*d* is the separation between the point force location along the $\hat{a}$ axis, *l* determines the position of the third point force location along $\hat{b}$ and *δ* is the Dirac delta function. Clearly, Eq (6) describes the tripod model but setting *β* = 0 reduces it to dipole model.

A simple calculation shows that the dipole moments of the force models are a function of the relative position between the point forces, *d* and *l*, and their strength, *α* and *β*,

$$\begin{array}{ccc} D_{aa} & = & -2\alpha d,\\ D_{bb} & = & -\beta l, \end{array}$$ (7)

where the off-diagonal components vanish. For keratocytes, *D*_*aa*_ is larger in magnitude than *D*_*bb*_ since 1) the cells are wider than longer (*d* > *l*) and 2) the traction force is stronger along the long cell axis (*α* > *β*). Finally, the force quadrupole components are given by,

$$\begin{array}{ccc} Q_{aab} & = & \beta d^{2},\\ Q_{bbb} & = & -\beta l^{2}, \end{array}$$ (8)

and the others vanish. Hence, the tripod model captures the fact that *Q*_*aab*_ is a large and positive component of the quadrupole force integral. It also implies, that *Q*_*bbb*_ is negative and smaller in magnitude, in agreement with the experimental results shown in Fig 3 and S1 Appendix. In fact, *Q*_*bbb*_ is always very small compared to the largest components. Hence, from now on, we will set *l* = 0 to describe a system of migrating keratocytes (this has no consequence for the dipole model where *β* = 0). Also note that the tripod model explains why *Q*_*aab*_ tends to be larger than *Q*_*aaa*_ even with traction forces along $\hat{a}$ much larger than those along $\hat{b}$. This means that the lateral component of the traction (along $\hat{a}$) does not break the symmetry of the perfect finite dipole. For perfect dipoles with *β* = 0, all quadrupole components vanish.

**Fig 5:**
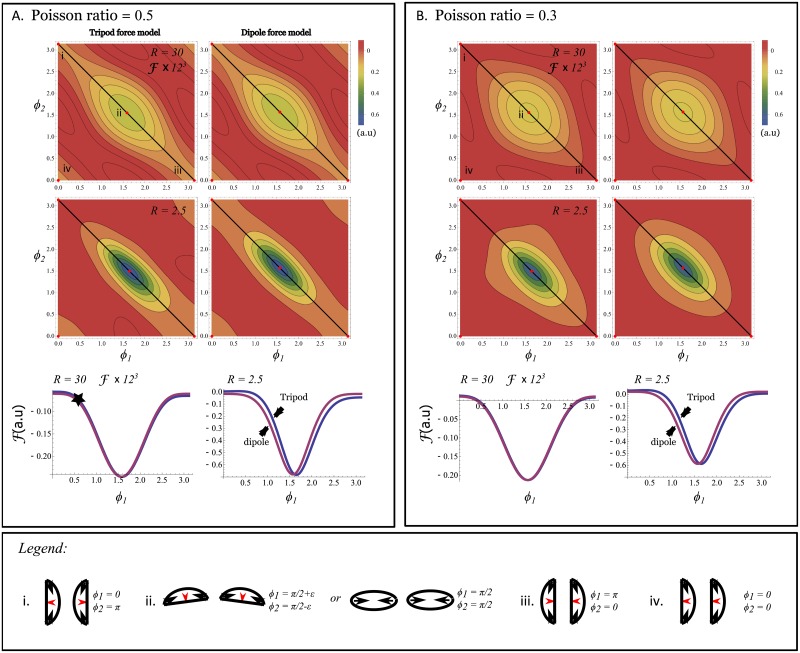
A. and B. show the interaction energy landscape, F given by Eq (1) in arbitrary units, between two tripods (left) and dipoles (right), both lying on the *x*-axis and separated by a distance *R* = 30*d* (top) and *R* = 2.5*d* (bottom). Note that the large separation interaction energy has been multiplied by 12^3^ to put it on the same scale as the one for short cell separation. Local extrema in the cell’s orientation space (*ϕ*_1_, *ϕ*_2_) are indicated by labels i, ii, iii and iv. The configurations associated with the four extrema are illustrated in the legend. A one-dimensional slice of the interaction energy is shown in the bottom plots for *ϕ*_2_ = *π* − *ϕ*_1_ (the diagonal line in the top contour plot that includes the first three extrema). The global minimum is the extremum labeled by ii; when two side-by-side cells point in the same direction for perfect dipoles, or when they are slightly tilted from each other for tripods. Panel A shows the results obtained with a fully incompressible substrate with Poisson ratio *σ* = 0.5, while the results shown in Panel B are obtained with *σ* = 0.3. Arbitrary units have been used for simplicity and because the response of the cells due to substrate-mediated interactions is controlled by another parameter (*ξ* in Eq (10)). Estimations of the latter parameter goes beyond the scope of the present study.

Linear elasticity theory is used to calculate the substrate mediated interaction energy between a pair of model cells. The result, given by Eq (B.1) in S1 Appendix, is used to generate the energy landscape described in Fig 5. The figure also compares results obtained with a finite dipole traction force model against the tripod model. The top contour plots in panels A and B show the interaction energy between two cells that lie on the *x*-axis as a function of their respective orientations (*ϕ*_1_ and *ϕ*_2_). In A, the Poisson ratio is set to *σ* = 0.5 (fully incompressible substrate) while in B, it is set to 0.3. The figure also shows where four extrema lie in the *ϕ*_1_-*ϕ*_2_ energy landscape. These correspond to the four orientations illustrated in the legend of the figure. Unsurprisingly, at large distances, the tripod and dipole force configurations predict the same preferred orientations between the cells. Also, the differences in energy between all configurations are small. When the distance between the two cells is reduced, the tripod and dipole force model have qualitative differences. First, at small distances (see Fig 5A that shows *R* = 2.5*d*), the symmetry about the *ϕ*_1_ = *ϕ*_2_ axis is clearly lost for tripods but it is preserved for dipoles. For cells as dipoles, the most energetically favorable configuration is when two side-by-side cells point in the same direction. For tripods, there is a small angle between the cells. For small cell size, this angle get be expressed as,

$$\begin{array}{c} \phi_{1}-\phi_{2}=\frac{6}{5-4\sigma }\frac{\beta }{\alpha }\frac{d}{R}+O{\left(\frac{d}{R}\right)}^{3}, \end{array}$$ (9)

which shows that the angle grows (and hence, the asymmetry about the *ϕ*_1_ = *ϕ*_2_ axis) with increasing Poisson ratio. Note that, for all distances and Poisson ratios, the global minimum is the extremum labeled by ii. The figure also shows a 2D plot of the *ϕ*_2_ = *π* − *ϕ*_1_ cut of the contour plot in the bottom part of panels A and B. It clearly illustrates the shift of the global minimum for tripods against dipoles. Further, it shows that the configuration labeled by i (cells directly pointing at one another) is a local minimum for tripods while it is a maximum for dipoles.

These findings allow us to illustrate the collision avoidance behavior for cells that approach each other in a nearly head-on manner. In other words, we will consider two cells that approach one another slowly from a configuration close the one labeled by i. More precisely, this initial configuration is defined by *ϕ*_1_ = *ϵ* and *ϕ*_2_ = *π* − *ϵ* for some small *ϵ*. For illustrative purposes, it is indicated by a star in the bottom plot of Fig 5A. If *ϵ* is large enough, cells that start in this initial configuration will immediately start to turn as they approach one another. The elastic interaction that drives that turn increases as the distance between the cells is reduced. The cells continue to turn until they reach configuration ii, in which case they move side by side if they are dipoles. On the other hand, they would continue to turn away from each other if they are tripods. In both cases, if the cells move slowly enough, the collision is avoided due to the substrate mediated cell-cell interactions. For dipoles, only initial configurations where *ϵ* is exactly zero would lead to a collision. For tripods, there is a small energy barrier going from configuration i to ii that, in theory, stabilizes configuration i to some degree. This means that there is a basin of attraction that would favor cells with initial configuration *ϕ*_1_ = *ϵ* and *ϕ*_2_ = *π* − *ϵ* with a small *ϵ* to collide.

Starting from an initial configuration as described by the other extrema, configurations iii and iv, do not lead to collision avoidance. From configuration iii, the cells will tend to migrate away from one another. Finally, a collision would occur between two cells that start from configuration iv, if the trailing cell moves faster than the leading one. There, the collision would not be avoided since an energy barrier needs to be crossed for the cells to turn and hence pass from configuration iv to ii. Further, such behavior was not observed in our experiments.

### Tripod force model: Dynamic considerations

We now extend our model for migrating keratocytes by adding simple dynamics. Our goal here is to highlight 1) in which dynamical regime the quasi-static analysis presented above is recovered and 2) what other types of migratory phenomena are predicted to emerge in other regimes. The results of this section are reported for the tripod model only.

We first assume that the dynamics of interacting tripods can be described by the following equations of motion,

$$\begin{array}{ccc} \frac{d\phi_{n}}{dt} & = & -\xi_{n}\frac{\partial F_{1,2}\left(\phi_{1},\phi_{2},x_{1}-x_{2}\right)}{\partial \phi_{n}}\\ \frac{dx_{n}}{dt} & = & V_{n}\hat{r}\left(\phi_{n}\right)+F_{\text{rep}}\left(\right|x_{1}-x_{2}\left|\right) \end{array}$$ (10)

where *ϕ*_*n*_ is the orientation of the cell velocity vector that represents the direction (and the short force component along $\hat{b}$) of cell *n*, **x**_*n*_ is the position of cell *n* and $F_{1,2}$ is the substrate mediated interaction energy given in S1 Appendix, where the dependence on angle and position have been made explicit. Note that we introduced a parameter *ξ* which is inversely proportional to the resistance of cells to reorient. Each cell *n* moves at a constant speed given by *V*_*n*_. The last term, **F**_rep_ in the last equation, is a short-range repulsive force that prevents the cell’s distance from becoming smaller than 2*d*. Note that our aim is to focus on the cell’s behavior before they touch. Hence, for simplicity, we did not include cell-cell adhesion.

There are two characteristic time scales in the above equation. The first can be defined as *t*_*ϕ*_ = *Ed*^2^/*αξ*, where *E* is the Young modulus of the substrate. It characterizes the time over which the cell will reorient as a result of the elastic force. The second is *t*_*x*_ = *d*/*V*. It characterizes the time scale for the cells forward motion. In what follows, we will vary these characteristic times by allowing the cells to move at different speeds and to react over different timescales in response to the elastic force.

**Fig 6:**
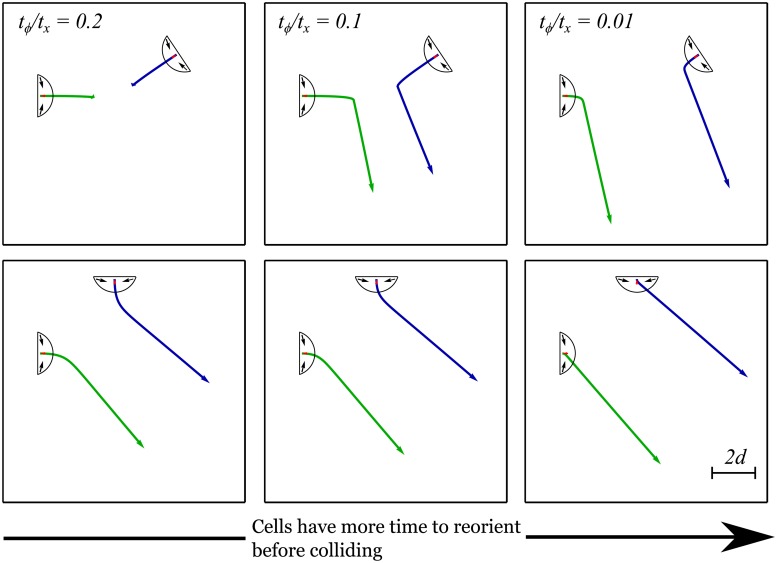
Effect of varying the two timescales that play a role in collision avoidance behavior. *t*_*ϕ*_ controls the time is takes for a cell to turn in the presence of a force and *t*_*x*_ controls the time it takes for a cell to move forward (see text). Examples of the simulated trajectories of two cells that would collide if they did not interact through the substrate. In all cases, Eq (10) is simulated with *ξ*_1(2)_ = *d* = *α* = *β* = *E* = 1. All units are arbitrary. The two cells have the same velocities which change from *V* = 0.2 (left) to *V* = 0.01 (right). The trajectories shown above differ solely due to the ratio of the two timescales, *t*_*ϕ*_/*t*_*x*_, which varies from 0.2 to 0.01. The top and bottom rows have different initial conditions for the position of the second cell (i.e. the cells start approaching one another with a different angle). Note that a collision occurs only in the top left panel. Movies of these simulations and of those obtained with other initial orientations are given in S5, S6 and S7 Videos.

We start by presenting the analysis of a series of trajectories where the cells would collide if they did not interact elastically. The results are shown in Fig 6 and all simulation parameters are given in the caption. In each case, we simulated 12 angles of approach ranging from −*π* to −3*π*/16. The figure shows −13*π*/16 (top panels) and −*π*/2 (bottom panels). The other angles of approach are shown as supplementary movies (see S5, S6 and S7 Videos). Further, note that in the absence of substrate-mediated interactions, the two cells would travel along a straight line and over a distance equal to 4*d* before colliding.

Fig 6 shows the effect of varying the ratio of the time scales defined above. We start by focusing on the top row of Fig 6, where the initial orientation of the cells is such as they are on their way to collide in a head-on manner. In the left column, *t*_*ϕ*_/*t*_*x*_ = 0.2, the separation of timescales is not sufficiently large to prevent the collision to occur (as shown in the top left panel). As the separation of time scale is increased to *t*_*ϕ*_/*t*_*x*_ = 0.1 (middle panel), and *t*_*ϕ*_/*t*_*x*_ = 0.01 (right panel), the cells have enough time to reorient, change their migratory course, and avoid the collision. Note that in the second row, no collision is observed for all values of *t*_*ϕ*_/*t*_*x*_. This occurs because the substrate-mediated interaction energy is stronger for those initial configurations, as shown in Fig 5.

**Fig 7:**
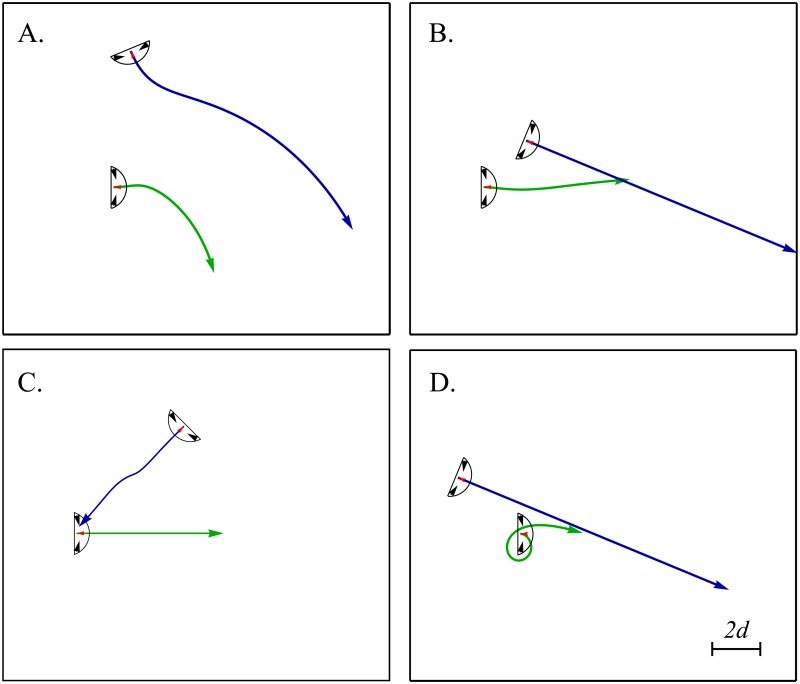
Non-identical cells: Examples of some effects that occur when the model cells properties are varied. Eq (10) is simulated with *d* = *α* = *β* = *E* = 1 (unless otherwise specified) and varying cell velocities, response to the elastic force and cells initial configurations. A. two cells that would collide approach one another at different speeds (*V*_1_ = 0.1, *V*_2_ = 0.2, *ξ*_1_ = *ξ*_2_ = 1). B-D. One of the two cells does not react to the elastic force. B. The cell that does not respond to the interaction force moves faster (*V*_1_ = 0.025, *V*_2_ = 0.05, *ξ*_1_ = 0.05 = *ξ*_2_ = 0, *β*_1,2_ = 0.5) and the slow cell follows it. C. The two cells move at the same speed. The cells would move toward one another but do not collide without the interaction. (*V*_1_ = 0.05, *V*_2_ = 0.05, *ξ*_1_ = 0 = *ξ*_2_ = 0.5, *β*_1,2_ = 0.5) D. A slower cell makes a loop as a faster, unresponsive cell passes it (*V*_1_ = 0.025, *V*_2_ = 0.05, *ξ*_1_ = 1 = *ξ*_2_ = 0). Movies of these simulations and of those obtained with other initial orientations are given in the Supporting Information.

We conclude this section by showing other types of cell pair trajectories that arise when the dynamical parameters, initial conditions and traction forces are varied. The results are shown in Fig 7. Part A gives an example of collision avoidance for cells that move at different speeds. The result is that after the cells scatter, they migrate away from each other with a small angle as in Fig 6 but in a curved manner. The faster cell moves along the path with the smallest curvature. Part B shows a cell pair trajectory that resembles the experimental one we reported in Fig 2B. In this case, both cells start with a similar orientation, one cell moves faster and does not respond to the interaction force. The other, slower trailing cell, turns in the wake of the trajectory of the first one. As the distance between the cells increases, the turning eventually stops. Part C shows a behavior that has similarities with Fig 2C. The two cells approach each other with nearly opposite orientations. One does not react to the interaction force, while the other turns away from the first cell when they get close. In the experiment, the turning cell turns away from the other but in the simulation, this occurs only transiently. After this transient event, it turns in the other direction, through the wake of the cell that moves in a straight line. We hypothesize that this difference may be due to the fact that the traction force pattern applied by the cell reorients on a longer timescale, and lags behind the velocity reorientation, as shown in Fig 3 and in the figure given in S1 Appendix. The last panel, part D, shows a cell that starts in front of a faster moving one that does not respond to the elastic force. The slower cell performs a loop before it ends up following the faster cell, in an configuration that minimizes the elastic interaction energy (configuration iv in Fig 5). We did not record a similar behavior from our experiments. Supplementary movies accompanying Fig 7 are given in S8, S9, S10 and S11 Videos.

## Conclusion

In this work, we combined traction force microscopy experiments using migrating keratocytes with a simple theory to qualitatively understand and explain the *collision avoidance behavior* observed experimentally. For cells that move toward another in a near head-on collision manner (see Fig 2B), we hypothesized that the cells will often turn and scatter without touching, due to substrate mediated elastic interactions. Our experiments show that the frequency of this phenomenon depends on the substrate stiffness: Fewer collisions are observed on the softer of the two substrate. The mechanism for collision avoidance that we propose is based on the premise that cells tend to reorient so that the energy stored in the substrate is minimized.

A minimum energy model was used to explain how cells could avoid collisions due to their interaction via the elastic substrate. More precisely, our model treated the cells as self-propelled traction force dipoles or tripods. Both descriptions of the traction force pattern account for the traction force dipoles (Eq (4)) that we observed experimentally. By construction, only the tripod model can account for the traction force quadrupole moments (Eq (5)). All migrating keratocytes that we observed had a large and non-trivial quadrupole moment (*Q*_*aab*_ in Fig 3). The minimum energy models we proposed showed how the elastic interaction energy tends to make the cells turn before they touch. The tripod model predicts that cells would then scatter at a finite angle. In the case of the dipole model, the cells would rather migrate side by side. We further showed that the quadrupole moment of the cell traction plays a role in determining the scattering angle between the two cell trajectories after the collision avoidance event (see Eq (9)).

In a recent study by Tanimoto et al. [23], the traction force quadrupole moment was also shown to play an important role in the migration direction of *Dictyostelium Discoideum*. Our investigations reinforce the importance of the quadrupole moment in the migratory behavior of cells on flexible substrates. Tanimoto et al. proposed that it plays a role at the level of single cell behavior. We further highlight the role the quadrupole moment can play on cell-cell substrate mediated elastic interactions and hence on the migratory behavior of cell groups.

The elastic modulus of the substrate plays an important role on the emergence of collision avoidance. Our experiments show that collision avoidance is observed on both the softer and stiffer surfaces. The main difference is that on the softer surfaces, traction stresses are transmitted further. In the case where the cells generate the same traction force on substrates of different stiffness, one can show that the substrate mediated interaction energy will be weaker on the stiffer substrate [19]. This can be shown by inspection of Eq (1) where, for constant **t**_1/2_, the energy is inversely proportional to the Young’s modulus. In other words, at any fixed cell-cell distance, the interaction between cells will be weaker on the stiffer substrate. As a result, if cell-cell interactions via the substrate plays a dominant role, we would expect collision avoidance to occur less frequently on the stiffer substrate. This is what we observed in our experiments.

Recent studies suggested that *traction* forces can be larger on stiffer substrates [25, 26]. If this is the case here, the effect would increase the substrate mediated *interaction* energy between cells on the stiff substrate. It could even be stronger on the stiffer substrate. However, for our system, the interaction energy between cells is stronger on the softer substrate (i.e., the bead displacement fields extend further away from the cells). Hence, any increase in traction force on the stiffer gel is not sufficient to overcome the decrease in interaction energy due to the larger modulus.

Our findings suggest that collision avoidance may act to enhance cell scattering during the early stages of wound healing by reducing the number of cell collisions. Other recent studies based on pairwise substrate mediated cell-cell interactions to explain emerging phenomena focused on cardiomyocytes synchronized beating [27] and angiogenesis [28]. For a recent review on the role of the extracellular matrix in cell behavior, see [29].

Note that the results reported in Figs 2 and 3 showed that the larger isolated cell control applied much larger forces on the substrate. The theory we used to describe the cell-cell substrate mediated interaction force remain valid if the two cells apply different traction forces. However, it may be that the larger cell will need a proportionally larger force to change its orientation due to the presence of the smaller cell. In other words, it is possible that the resistance to reorientation, 1/*ξ* in Eq 10, varies with cell size and overall cell traction force magnitude. Providing a mechanism for *ξ* goes beyond the scope of our study.

It is important to note that our minimum energy model uses a static pattern of the traction forces exerted by the cells. In other words, we have shown that it is not necessary to include changes in the magnitude and direction of local traction forces to explain the emergence of collision avoidance and the observed finite scattering angles between cells. However, it is known that focal adhesions are dynamic and that they can be mechano-sensitive [30]. Therefore, collision avoidance might be enhanced from force-induced adhesion disassembly at opposing edges of neighboring cells, causing them to turn away in opposite directions. In support of this possibility, our experiments showed that while the keratocytes turned, the left-right symmetry of the traction force pattern was broken. This is one way to quantify mechano-sensitivity and it is shown on the third row of Fig 3 which gives the distribution of the angle between the small force dipole and the short cell axis. The results shown are for the two cells involved in collision-avoidance behavior in Fig 2A and the isolated cell control in Fig 2D. We further showed in the second row of Fig 3 that the cells principal dipole orientation lags behind the change in cell velocity direction. This is clearly seen for the two cells that turn to avoid each other where the small dipole component of the traction force reorients slower than the cell velocity. Both this result and the left-right symmetry breaking just described were similarly observed for the other cell pair trajectories reported in Fig 2B and 2C, as shown in S1 Appendix.

This last result may appear contradictory because the only way the cell can move is by applying a net force on the substrate. However, as just mentioned, cell direction changes *before* the orientation of the dipole components of the traction force. This is due to the fact that the cell velocity is, up to a proportionality constant, equal to the traction force *monopole*. This component is orders or magnitude smaller than the higher multipoles moment. Hence, we assumed that it could be neglected in the traction force analysis (i.e. it is not strong enough to change the long range substrate deformation and the substrate mediated cell-cell interactions). The fact that the cell velocity reorients faster than the force dipole moments can be explained as follows. The local traction forces applied by each cell sum up to a non-zero force monopole which is unobservable by traction force microscopy but that reorients more easily than the larger traction forces that dominate the higher multipole traction force components.

To conclude, we showed that a variety of cell pair trajectories can arise by extending our model to include dynamics. The results (Figs 6 and 7) showed that, with the right dynamical parameters, experimentally observed cell pair trajectories can be reproduced. It would be worthwhile investigating collective effects, as is done in studies of flocking [31–33], as the elastic effects we have identified would appear to provide a mechanism for long-range interactions between migrating cells. The substrate elastic energy minimization principle that we used in this study and initially proposed by others elsewhere (see [19] and references therein) could provide an alternative mechanism for collective motion emerging for cells on flexible substrates.

## Supporting information

S1 Appendix Supplementary appendix. Contains extra details on the substrate elasticity characterization, extra theoretical details and the multipole analysis for trajectories in Fig 2B and 2C.
(PDF)

S1 Video Supplementary movie 1. Movie accompanying Fig 2A.
(MOV)

S2 Video Supplementary movie 1. Movie accompanying Fig 2B.
(MOV)

S3 Video Supplementary movie 3. Movie accompanying Fig 2C.
(MOV)

S4 Video Supplementary movie 4. Movie accompanying Fig 2D.
(MOV)

S5 Video Supplementary movie 5. Movie accompanying Fig 6 left panels.
(MOV)

S6 Video Supplementary movie 6. Movie accompanying Fig 6 middle panels.
(MOV)

S7 Video Supplementary movie 7. Movie accompanying Fig 6 right panels.
(MOV)

S8 Video Supplementary movie 8. Movie accompanying Fig 7A.
(MOV)

S9 Video Supplementary movie 9. Movie accompanying Fig 7B.
(MOV)

S10 Video Supplementary movie 10. Movie accompanying Fig 7C.
(MOV)

S11 Video Supplementary movie 11. Movie accompanying Fig 7D.
(MOV)
