# Supplementary material for: Substrate mediated interaction between pairs of keratocytes: Multipole traction force models describe their migratory behavior
Source: PLoS One. 2019 Mar 1;14(3):e0212162. doi: 10.1371/journal.pone.0212162 (PMC6396918; doi:10.1371/journal.pone.0212162)
Supplement: S1 Appendix — Contains extra details on the substrate elasticity characterization, extra theoretical details and the multipole analysis for trajectories in Fig 2B and 2C. (PDF) [file pone.0212162.s001.pdf]

Appendix for "Substrate mediated interaction between pairs of keratocytes: Multipole traction force models describe their migratory behavior."

Benoit Palmieri,[1] Christine Scanlon,[2] Daniel Worroll III,[2] Martin Grant,[1] and Juliet Lee[2,*]

[1] *Department of Physics, McGill University, 3600 University, Montréal, Québec, Canada H3A 2T8*

[2] *Department of Molecular & Cell Biology, 91 North Eagleville Road, Unit 3125, BSP 306, Storrs, CT 06269-3125*

(Dated: January 7, 2019)

APPENDIX A: MEASUREMENT OF SUBSTRATE STIFFNESS

The Elastic modulus of the gelatin substrata was measured using the bead indentation method as described previously [1]. Briefly, measurements of bead indentation in the gelatin substrata were taken at 5 different locations and the average value was used to calculate the Elastic modulus using the Hertz equation [2] below:

$$E = \frac{3(1-\sigma^2)f}{4r^{1/2}\delta^{3/2}} \tag{A.1}$$

where r is the bead radius, f is the force applied by the steel bead, δ is the indentation of the substratum, and σ is the Poisson ratio of the substrate.

The results are shown in Fig. S.1.

APPENDIX B: OTHER LARGE QUADRUPOLE COMPONENTS

Here, we will show how Q_{aba} and Q_{aaa}, the other large quadrupole components that are observed experimentally, are simple extensions of the two point finite force dipole. In fact, in the integrals given by Eqs. (4) and (5) in the main text, the origin is the cell center. Because the traction, integrated over the cell area, is enforced to vanish, this has no consequence on the dipole calculation. However, this is not true for the quadrupole. Hence, we will consider the large force dipole part of Eq. (6) in a coordinate system where the cell center does not correspond to the origin

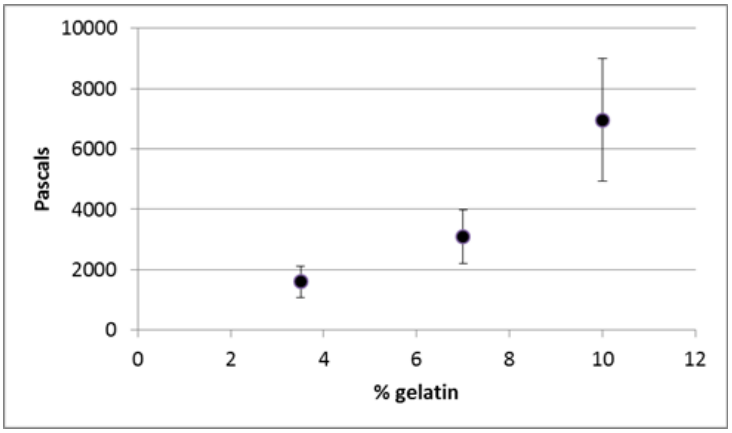

% gelatin	Mean (Pa)	Standard Deviation
3.5	1595	519
7	3093	899
10	6955	2034

FIG. S.1: The graph and table show the mean and Standard Deviation of the Elastic modulus obtained from calibration experiments with gelatin gels composed of 3.5, 7 and 10 percent gelatin. Each data point was obtained from the averaged measurements taken from 7 substrata for each concentration of gelatin. Note that although there is some variation in these measurements, there is no overlap in the range of measurements between 3.5 and 10 percent gelatin.

*Correspondence to juliet.lee@uconn.edu

in Eq. (6)

$$\mathbf{t}_d = \hat{a}\left(\alpha\delta(\mathbf{x} - \boldsymbol{\Delta} + \hat{a}d) - \alpha\delta(\mathbf{x} - \boldsymbol{\Delta} - \hat{a}d)\right), \tag{B.1}$$

where $\boldsymbol{\Delta}$ represents the difference between the two coordinate systems. This shift has no effect on the principal dipole components. However, the quadrupole becomes,

$$\begin{aligned} Q_{aaa} &= -4\alpha d\Delta_a, \\ Q_{aba} &= -2\alpha d\Delta_b, \end{aligned} \tag{B.2}$$

where all other components are zero. This explains how a difference between the cell center (defined by the cell contour) and the center of symmetry of the forces, leads to non-zero quadrupole moments, even with only two point forces. Since these two point forces are dominated by their dipole moments, their quadrupole contribution is neglected.

Interestingly, Tanimoto et al. [3] observed a large Q_{aaa} quadrupole component in the force distribution of migrating Dictyostelium. In their study, this arises because $\mathbf{t}(a,b) \neq \mathbf{t}(-a,b)$ and a is the direction of motion. In our study, the cells move along b. Hence, one may expect Q_{bbb} to be non-zero due to force distribution asymmetry along the b-axis. The forces along b are relatively small for keratocytes and hence, Q_{bbb} is a minor quadrupole component. However, they may contribute to the strength of the Q_{aba} component. Similarly, there may be some degree of asymmetry in the force distribution along a which can also contribute to Q_{aaa}, in addition to the effect described above based on the location of the cell center.

APPENDIX C: INTERACTION ENERGY BETWEEN A PAIR OF TRIPOLES

The substrate mediated interaction energy between a pair of cells modeled as tripoles is obtained by Eq. (6) for $\mathbf{t}_1$ and $\mathbf{t}_2$ in Eq. (1) in the main text. The result is

$$\mathcal{F}_{1,2} = \frac{1+\sigma}{\pi E}\left((1-\sigma) f_{1,2}^{(a)} + \sigma f_{1,2}^{(b)}\right), \tag{C.1}$$

where

$$\begin{aligned} f_{1,2}^{(a)} = &\frac{\beta^2\cos(\phi_1-\phi_2)}{r} \\ &- \frac{[\beta^2\cos(\phi_1-\phi_2) - 2\alpha\beta\sin(\phi_1-\phi_2)]}{2[(x-d\sin\phi_1)^2 + (y+d\cos\phi_1)^2]^{1/2}} - \frac{[\beta^2\cos(\phi_1-\phi_2) + 2\alpha\beta\sin(\phi_1-\phi_2)]}{2[(x+d\sin\phi_1)^2 + (y-d\cos\phi_1)^2]^{1/2}} \\ &- \frac{[\beta^2\cos(\phi_1-\phi_2) - 2\alpha\beta\sin(\phi_1-\phi_2)]}{2[(x-d\sin\phi_2)^2 + (y+d\cos\phi_2)^2]^{1/2}} - \frac{[\beta^2\cos(\phi_1-\phi_2) + 2\alpha\beta\sin(\phi_1-\phi_2)]}{2[(x+d\sin\phi_2)^2 + (y-d\cos\phi_2)^2]^{1/2}} \\ &+ \frac{(4\alpha^2+\beta^2)\cos(\phi_1-\phi_2)}{4[(x+d\sin\phi_1-d\sin\phi_2)^2 + (y-d\cos\phi_1+d\cos\phi_2)^2]^{1/2}} \\ &+ \frac{(4\alpha^2+\beta^2)\cos(\phi_1-\phi_2)}{4[(x-d\sin\phi_1+d\sin\phi_2)^2 + (y+d\cos\phi_1-d\cos\phi_2)^2]^{1/2}} \\ &- \frac{(4\alpha^2-\beta^2)\cos(\phi_1-\phi_2) + 4\alpha\beta\sin(\phi_1-\phi_2)}{4[(x-d\sin\phi_1-d\sin\phi_2)^2 + (y+d\cos\phi_1+d\cos\phi_2)^2]^{1/2}} \\ &- \frac{(4\alpha^2-\beta^2)\cos(\phi_1-\phi_2) - 4\alpha\beta\sin(\phi_1-\phi_2)}{4[(x+d\sin\phi_1+d\sin\phi_2)^2 + (y-d\cos\phi_1-d\cos\phi_2)^2]^{1/2}}, \end{aligned} \tag{C.2}$$

and

$$
\begin{aligned}
f^{(b)}_{1,2} &= \frac{\beta^2}{r^3}\left[x\cos\phi_1 + y\sin\phi_1\right]\left[x\cos\phi_2 + y\sin\phi_2\right] \\
&- \frac{\beta[2\alpha d + (\beta x + 2\alpha y)\cos\phi_1 + (\beta y - 2\alpha x)\sin\phi_1]}{2[(x - d\sin\phi_1)^2 + (y + d\cos\phi_1)^2]^{3/2}}\left[x\cos\phi_2 + y\sin\phi_2 - d\sin(\phi_1 - \phi_2)\right] \\
&- \frac{\beta[2\alpha d + (\beta x - 2\alpha y)\cos\phi_1 + (\beta y + 2\alpha x)\sin\phi_1]}{2[(x + d\sin\phi_1)^2 + (y - d\cos\phi_1)^2]^{3/2}}\left[x\cos\phi_2 + y\sin\phi_2 + d\sin(\phi_1 - \phi_2)\right] \\
&- \frac{\beta[-2\alpha d + (\beta x - 2\alpha y)\cos\phi_2 + (\beta y + 2\alpha x)\sin\phi_2]}{2[(x - d\sin\phi_2)^2 + (y + d\cos\phi_2)^2]^{3/2}}\left[x\cos\phi_1 + y\sin\phi_1 + d\sin(\phi_1 - \phi_2)\right] \\
&- \frac{\beta[-2\alpha d + (\beta x + 2\alpha y)\cos\phi_2 + (\beta y - 2\alpha x)\sin\phi_2}{2[(x + d\sin\phi_2)^2 + (y - d\cos\phi_2)^2]^{3/2}}\left[x\cos\phi_1 + y\sin\phi_1 - d\sin(\phi_1 - \phi_2)\right] \\
&+ \frac{[2\alpha d + (\beta x - 2\alpha y)\cos\phi_1 - 2\alpha d\cos(\phi_1 - \phi_2) + (2\alpha x + \beta y)\sin\phi_1 + \beta d\sin(\phi_1 - \phi_2)]}{4[(x + d\sin\phi_1 - d\sin\phi_2)^2 + (y - d\cos\phi_1 + d\cos\phi_2)^2]^{3/2}} \\
&\qquad \times \left[(x + d\sin\phi_1 - d\sin\phi_2)(\beta\cos\phi_2 + 2\alpha\sin\phi_2) + (y - d\cos\phi_1 + d\cos\phi_2)(\beta\sin\phi_2 - 2\alpha\cos\phi_2)\right] \\
&+ \frac{[2\alpha d + (\beta x + 2\alpha y)\cos\phi_1 - 2\alpha d\cos(\phi_1 - \phi_2) - (2\alpha x - \beta y)\sin\phi_1 - \beta d\sin(\phi_1 - \phi_2)]}{4[(x - d\sin\phi_1 + d\sin\phi_2)^2 + (y + d\cos\phi_1 - d\cos\phi_2)^2]^{3/2}} \\
&\qquad \times \left[(x - d\sin\phi_1 + d\sin\phi_2)(\beta\cos\phi_2 - 2\alpha\sin\phi_2) + (y + d\cos\phi_1 - d\cos\phi_2)(\beta\sin\phi_2 + 2\alpha\cos\phi_2)\right] \\
&+ \frac{[2\alpha d + (\beta x + 2\alpha y)\cos\phi_1 + 2\alpha d\cos(\phi_1 - \phi_2) - (2\alpha x - \beta y)\sin\phi_1 + \beta d\sin(\phi_1 - \phi_2)]}{4[(x - d\sin\phi_1 - d\sin\phi_2)^2 + (y + d\cos\phi_1 + d\cos\phi_2)^2]^{3/2}} \\
&\qquad \times \left[(x - d\sin\phi_1 - d\sin\phi_2)(\beta\cos\phi_2 + 2\alpha\sin\phi_2) + (y + d\cos\phi_1 + d\cos\phi_2)(\beta\sin\phi_2 - 2\alpha\cos\phi_2)\right] \\
&+ \frac{[2\alpha d + (\beta x - 2\alpha y)\cos\phi_1 + 2\alpha d\cos(\phi_1 - \phi_2) + (2\alpha x + \beta y)\sin\phi_1 - \beta d\sin(\phi_1 - \phi_2)]}{4[(x + d\sin\phi_1 + d\sin\phi_2)^2 + (y - d\cos\phi_1 - d\cos\phi_2)^2]^{3/2}} \\
&\qquad \times \left[(x + d\sin\phi_1 + d\sin\phi_2)(\beta\cos\phi_2 - 2\alpha\sin\phi_2) + (y - d\cos\phi_1 - d\cos\phi_2)(\beta\sin\phi_2 + 2\alpha\cos\phi_2)\right],
\end{aligned}
\tag{C.3}
$$

where cell 1 is at the origin and cell 2 is at $\mathbf{r} = \hat{x}x + \hat{y}y$ and equivalently, $x = r\cos\theta$ and $y = r\sin\theta$. The orientation of cells 1 and 2 are determined by $\phi_{1,2}$ which are both defined to be zero if the orientation vector points along the positive x-axis.

In the limit of small separation between the point force ($d \to 0$)and for $\beta = 0$, the above expression reduces to,

$$
\begin{aligned}
&\mathcal{F}^{(d)}_{1,2} = \\
&\quad -\frac{d^2(1+\sigma)\alpha^2}{2\pi E r^3}\left[((2-\sigma)\cos 2(\phi_1 - \phi_2) + 15\sigma\cos 2(\phi_1 + \phi_2 - 2\theta) + 2(\sigma - 1)(3\cos 2(\phi_1 - \theta) + 3\cos 2(\phi_2 - \theta) - 1)\right] \\
&\quad +O(d^4)
\end{aligned}
\tag{C.4}
$$

a result which was first derived in Ref. [4].

[1] M. Frey, A. Engler, J. Lee, Y.-L. Wang, and D. Discher, Methods Cell Biol. **83**, 47 (2007).
[2] H. Hertz, J. Reine Angew. Mathematik **92**, 156 (1882).
[3] H. Tanimoto and M. Sano, Biophys. J. **106**, 16 (2014).
[4] I. Bischofs, S. Safran, and U. Schwarz, Phys. Rev. E **69**, 021911 (2004).

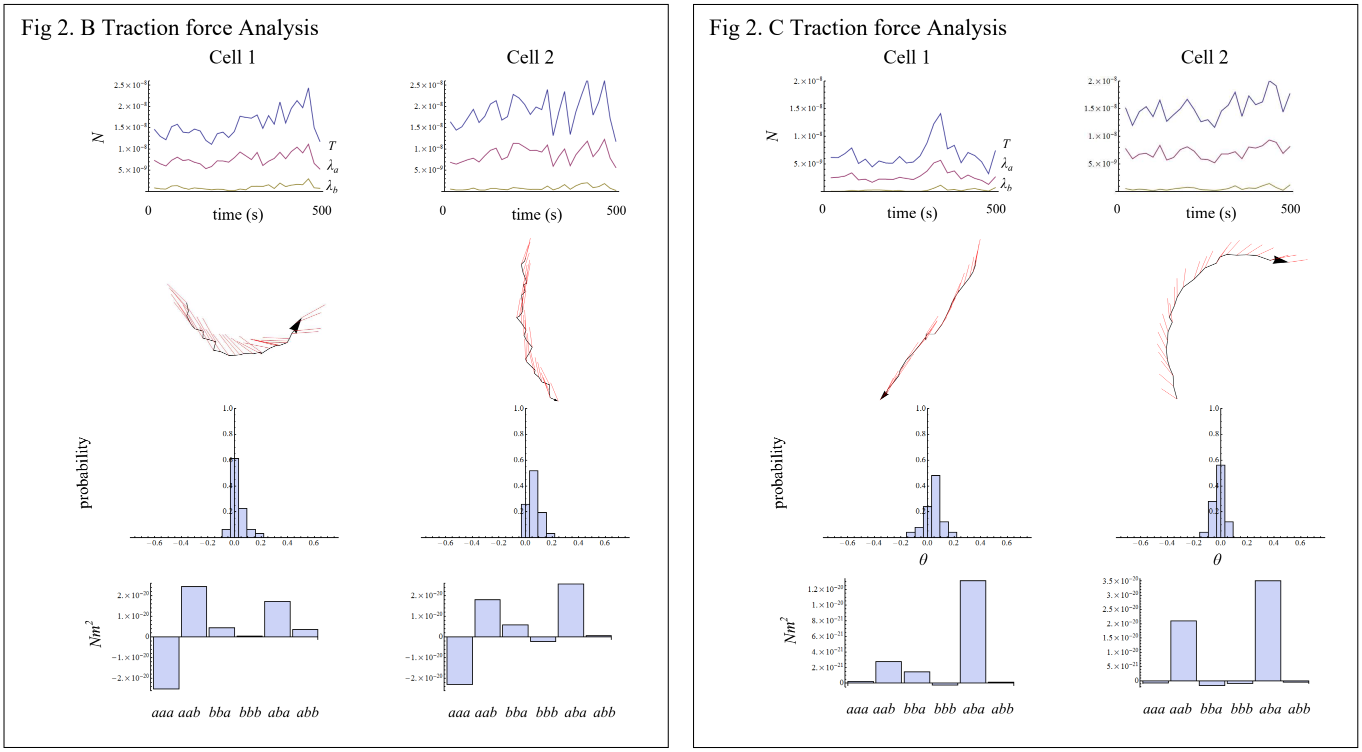

FIG. S.2: Traction force analysis of the two interacting cells shown in Fig. 2 B (left box) and C (right box). The meaning of all panels is the same as in Fig. 3 in the main text
